# Inhalations in the Treatment of Diseases of the Respiratory Passages, Particularly as Affected by the Use of Atomized Fluids

**Published:** 1867-05

**Authors:** 


Inhalations in the Treatment of Diseases of the Respiratory Passages, particularly as affected by the use of atomized fluids. By J. M. DaCosta, M.D., Physician to Pennsylvania Hospital; Fellow of College of Physicians and Surgeons, etc., etc. Philadelphia: J. B. LIPPINCOTT & Co. 1867.

This is a monograph of 86 pages, very neatly printed and bound. It contains five chapters on the following topics:

1st. The History of Inhalations and the Apparatus Employed.

2d. The Mode of Administering Inhalations.

3d. The Penetrability of Atomized Fluids into the Air-Passages.

4th. Doses of Medicine for Inhalations.

5th. Therapeutic Considerations.

It is a work of real value. For sale by S. C. GRIGGS & Co., 148 Lake St.

